# Radioprotectors.org: an open database of known and predicted radioprotectors

**DOI:** 10.18632/aging.103815

**Published:** 2020-08-15

**Authors:** Alexander M. Aliper, Marine E. Bozdaganyan, Viktoria A. Sarkisova, Alexander P. Veviorsky, Ivan V. Ozerov, Philipp S. Orekhov, Mikhail B. Korzinkin, Alexey Moskalev, Alex Zhavoronkov, Andreyan N. Osipov

**Affiliations:** 1 Insilico Medicine, Hong Kong Science and Technology Park, Hong Kong; 2 Lomonosov Moscow State University, School of Biology, Moscow, Russia; 3 N.N. Semenov Federal Research Center for Chemical Physics, Russian Academy of Sciences, Moscow, Russia; 4 The Moscow Institute of Physics and Technology, Moscow Region, Dolgoprudny, Russia; 5 Department of Radioecology, Laboratory of Geroprotective and Radioprotective Technologies, Institute of Biology of the FRC of Komi Science Center, Ural Branch, Russian Academy of Sciences, Syktyvkar, Komi Republic, Russia; 6 State Research Center-Burnasyan Federal Medical Biophysical Center of Federal Medical Biological Agency (SRC-FMBC), Moscow, Russia

**Keywords:** radioprotectors, radiation mitigators, ionising radiation, antioxidants, free radical scavengers

## Abstract

The search for radioprotectors is an ambitious goal with many practical applications. Particularly, the improvement of human radioresistance for space is an important task, which comes into view with the recent successes in the space industry. Currently, all radioprotective drugs can be divided into two large groups differing in their effectiveness depending on the type of exposure. The first of these is radioprotectors, highly effective for pulsed, and some types of relatively short exposure to irradiation. The second group consists of long-acting radioprotectors. These drugs are effective for prolonged and fractionated irradiation. They also protect against impulse exposure to ionizing radiation, but to a lesser extent than short-acting radioprotectors. Creating a database on radioprotectors is a necessity dictated by the modern development of science and technology. We have created an open database, Radioprotectors.org, containing an up-to-date list of substances with proven radioprotective properties. All radioprotectors are annotated with relevant chemical and biological information, including transcriptomic data, and can be filtered according to their properties. Additionally, the performed transcriptomics analysis has revealed specific transcriptomic profiles of radioprotectors, which should facilitate the search for potent radioprotectors.

## INTRODUCTION

Among the tasks of modern radiobiology [1], searching for the agents with radioprotective action is one of the most important. Such activity can be achieved by using gene therapy for increasing radioresistance by exogenous engineered DNA repair and radioprotective constructs, replacing organic molecules with strengthened isoforms, slowing down metabolic activity while maintaining cognitive function or strengthening the regulation of endogenous repair and radioprotective machinery by means of chemical compounds. Only two radioprotective compounds, amifostine, and palifermin, currently have the US FDA approval for use in radiation therapy. However, several agents have been reported that show therapeutic promise [2]. Creating a database on radioprotectors is a necessity dictated by the modern development of science and technology.

The success in the development of radioprotective agents depends on an understanding of the molecular biology of radiation damage [3]. Increasing the radioresistance of the different tissues can be achieved with procedures that affect the primary radiochemical reactions, the protective mechanisms of the organism itself, or both.

All radiation modificator agents [4] can be divided into two groups: radiation mitigators (or simply mitigators) and radioprotectors (radioprotective agents). Radiation mitigators are substances which are used after irradiation that can reduce the negative effect of radiation. Radiation mitigators include, for example, substances such as TGF-β receptor inhibitors, protease inhibitors, COX2 inhibitors, and others [5]. Thus, radiation mitigators neutralize the negative consequences of mitotic cell death and DNA damage, reduce the activity of cytokine cascades, reducing the level of vascular damage, tissue hypoxia, and fibrosis [6].

In contrast, radioprotectors are drugs or compositions of drugs that are injected into the body before it is irradiated in order to provide a high protective effect. Radioprotectors are chemical compounds obtained synthetically or extracted from natural products. Their protective effect is manifested by a smaller lesion during the irradiation of radiosensitive tissues and their more rapid post-radiation recovery, which generally leads to a decrease in the severity of radiation injury. The use of radioprotectors after irradiation is usually ineffective [7, 8].

In this paper, we describe a manually curated database Radioprotectors.org containing an up-to-date list of substances with proven radioprotective properties at different levels of structural organization of the organisms.

## RESULTS AND DISCUSSION

**Figure 1:**
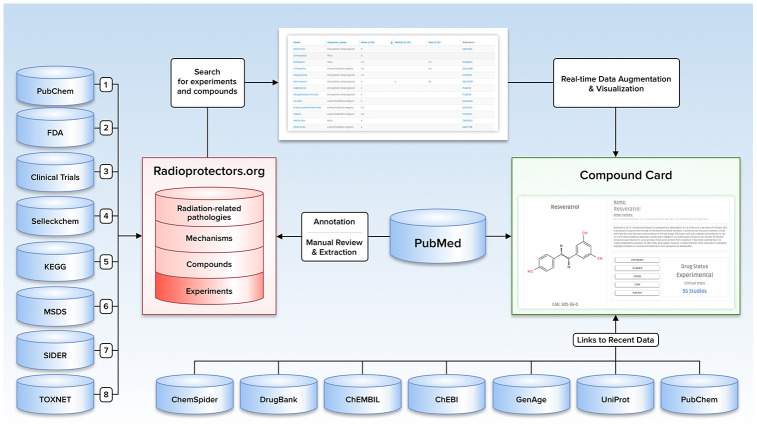
**Illustration depicting the content, data sources, and user-directed flow of Radioprotectors.org.**

The motivation behind the creation of the Radioprotectors database was to provide a one-stop resource for researchers interested in quick access to the results of experiments and approved drugs. As a result, a platform for cross-species, cross-study comparison of the effects of these compounds was created. The interface was developed to make it visually appealing and intuitive for rapid, effortless overviews of radioprotective compounds, with links to original studies and other databases for users seeking further detail. The site was not designed as a mere list of radioprotectors; instead, a comprehensive intervention profile was created for each compound, including its biochemistry and bioactivity, possible or known mechanisms of action, MESH indication, and its current drug status. Figure 1 represents a visual overview of the content, data sources, and user-directed exploration of these within Radioprotectors.

### Analysis of experiments related to radioprotective compounds

The database contains summaries of more than 150 radioprotective compounds. Each compound was manually selected from the existing biomedical literature by searching the PubMed database (http://www.ncbi.nlm.nih.gov/pubmed), DrugBank database (https://www.drugbank.ca/), and PubChem database (https://pubchem.ncbi.nlm.nih.gov/) using keywords relevant to pharmacological interventions in radioprotection.

All entries in Radioprotectors have links to original publications, making access to raw data fast and convenient. For any given compound, links to relevant study can be accessed directly from the search results or within each compound profile.

### Comparison with existing databases of radioprotectors and radiation mitigators

To date, there is only one database on radioprotectors [9], similar to that presented by the Bioinformatics Database of Radiosensitizers and Radioprotectors by the University of Mumbai. (http://bioph.mu.ac.in/Welcome/). DB includes about 100 compounds, a significant part of which is an extract of various plants, in which, as a rule, it is impossible to identify in its pure form a substance that can have a radioprotective effect. Radioprotectors.org includes more than 150 substances of both synthetic and natural origin and contains detailed information on the mechanism of action and pharmacological properties of the substance. Information on experimentation and efficiency is taken from peer-reviewed scientific journals. All substances have a unique identifier that allows one to quickly find the desired compound in leading chemical databases, including PubChem, ZINC, etc.

### Analysis of compounds with radioprotective activity

To date, all radioprotective drugs can be divided into two large groups, differing in their effectiveness depending on the type of exposure. The first of these is radioprotectors, highly effective for pulsed, and some types of relatively short exposure. These are radioprotectors mainly of short duration. Their protective activity, depending on the properties and methods of application, manifests itself within a few minutes or a maximum by the end of the first hour after administration, but is limited to 30 min-5 hours. In radioprotectors of this group, the highest level of the protective effect is usually observed when they are administered in maximum tolerated doses, which cause changes in the metabolism of radiosensitive cells. The second group consists of long-acting radioprotectors. These drugs are effective for prolonged (prolonged) and fractionated (fractional) irradiation. They also protect against impulse exposure to ionizing radiation, but to a lesser extent than short-acting radioprotectors. The duration of the protective action of radioprotectors of prolonged action can be from one up to several days. The radioprotective effect of these drugs is mainly associated with the mechanisms of increasing the general nonspecific resistance of the organism [10].

Short-acting radioprotectors, depending on the initial protective action mechanisms and chemical structure, are divided into the following groups [11, 12]: reducing agents, which include sulfur-containing compounds (cysteine, cystamine, cystaphos, etc.), antioxidants (ascorbic acid, vitamin E, tocopherols, etc.); and drugs that cause hypoxia of cells and tissues (indolealkylamines, methemoglobin formers, cyanides, azides, nitrites, etc.).

Sustained-release radioprotectors include drugs with anabolic properties (primarily with estrogenic activity), polyanionic polymers (heparin, chondroitin sulfate, and other polysaccharides, nucleic acids, polynucleotides and their derivatives, some vaccines, synthetic polymers).

The following mechanisms of radioprotectors action are possible [13, 14]:

- competition for strong oxidizing agents and free active radicals formed during irradiation of tissues and especially during radiolysis of water (peroxide or hydroperoxide radicals);
- increase in the content of endogenous thiol compounds in tissues;
- the formation of mixed disulfides and their temporary reversible bond;
- formation of temporary reversible bonds with radiosensitive groups of vital enzymes or other protein molecules, which ensures their protection at the time of irradiation;
- formation of strong compounds with heavy metals, providing accelerated course of chain oxidation reactions;
- migration of excess energy from the macromolecule to the radioprotector;
- inhibition of oxidation chain reactions;
- absorption of secondary ultraviolet radiation, exciting macromolecules such as nucleic acids;
- increase the stability and mobility of the protective mechanisms of the body, including compounds with the hormetic effect [15–18];
- inhibition of metabolism;
- detoxification or accelerated elimination of toxic products from the irradiated organism.

However, there is no such chemical substance, which would have all the above properties. That is why radioprotectors belong to the most diverse classes of chemical compounds.

Many of these agents are free radical scavengers/antioxidants. Superoxide dismutase and superoxide dismutase mimetics, nitroxides, and dietary antioxidants are all being investigated. Recently, alternative strategies of drug development have been evolving [19], which focus on targeting the series of cellular insult recognition/repair responses initiated after radiation. These agents, which include cytokines/ growth factors, angiotensin-converting enzyme inhibitors, and apoptotic modulators, show promise of having a significant impact on the mitigation of radiation injury [2].

### Antioxidants and free radical scavengers

Ionizing radiation induces damage of cellular structures in two primary ways: direct damage to DNA and generation of free radical-containing reactive molecules. Free radicals are generated through the interactions between ionizing radiation and small oxygen-containing molecules (including water). Reactive oxygen species (ROS) and reactive nitrogen species (RNS) are the main sources of damage to cell macromolecules. Ionizing radiation leads to the generation of ROS and RNS in the presence of oxygen and nitrogen. ROS include superoxide anion (O2•-), hydrogen peroxide (H2O2) and hydroxyl radical (OH•). Reactive forms of nitrogen are nitric oxide (NO•) and peroxynitrite (ONOO-) [20]. Free radicals that are generated by ionizing radiation can react with DNA, lipid membranes, and proteins causing damage and/or dysfunction to various cellular structures. The cell has mechanisms to mitigate and manage damage from free radicals. Hydroxide ions are reduced by the enzyme glutathione peroxidase and superoxide ions are reduced to hydrogen peroxide by superoxide dismutase. Hydrogen peroxide generated by superoxide dismutase is used by catalase to generate water. Significant damage to cellular structures occurs when the ionizing radiation-induced generation of radicals out-paces the cell’s ability to clear these reactive molecules [13, 21, 22].

Several approaches have been followed in recent decades to scavenge radicals [13, 21]. Sulfhydryl compounds, particularly the aminothiols and phosphorothioates contain an SH group, make them suitable for free radical scavenging because of their propensity to donate a hydrogen atom for the reduction of radical species [23]. We have included several substances including cysteine, cysteamine, glutathione, AET, amifostine [24]. Currently, amifostine is the only cytoprotective agent that is approved by the US FDA specifically for use as a radioprophylactic. The mechanism underlying amifostine’s protective action appears to be multifaceted, involving free radical scavenging, enhanced DNA protection and repair, and induction of hypoxia [25].

Redox homeostasis within a cell is maintained in part by a series of antioxidant enzymes that include glutathione peroxidase, catalase, and superoxide dismutase (SOD). All the SOD isoforms have been reported to have radioprotective potential, reducing acute radiation toxicity through neutralization of radiation-induced ROS and delaying radiation injury through suppression of chronic oxidative stress [26]. SOD mimetics have a metal ion (Cu, Fe, Mn, and Zn) at their active centers, which behave like the metal center of the SOD molecule. Advantages of the SOD-mimetics class of compounds include prolonged half-lives and widened time windows of action compared to native SOD. For example, M40403, manganese (Mn)-containing biscyclohexylpyridine, that has demonstrated equivalent or superior catalytic activity to that of native SOD, has been given FDA approval [27]. Also, this group includes AEOL 10150 [28] and Mn complexes EUK-189 and EUK-207 [29], tempol (4-hydroxy2,2,6,6-tetramethylpiperidine-1-oxyl) [29, 30].

A number of naturally occurring vitamins and dietary antioxidants have been tested for their efficacy as radioprotectors [31]. Both vitamin E and selenium, as well as their combination, have been reported to reduce radiation-induced transformations *in vitro* [32]. Vitamins C and E have been shown to decrease chromosomal damage, mutations and apoptosis in mammalian cells, and vitamin A and N-acetylcysteine have been suggested to be effective against radiation-induced carcinogenesis [33]. *In vivo* studies also report the use of antioxidants as effective radiation protectors. α-Lipoic acid significantly increased the survival rate following lethal total body irradiation in mice, while vitamins A, C, E and β-carotene have been shown to increase resistance to high doses of radiation, and *in vivo* protection against radiation-induced oxidative stress has been reported for L-selenomethionine and such antioxidants such as vitamins C and E, glutathione, α-lipoic acid, N-acetylcysteine and co-enzyme Q10 [34]. Another naturally occurring antioxidant receiving considerable interest is the hormone melatonin and its analogs, which have been documented to have a radioprotective effect in normal tissues in a number of animal models while, at the same time, exert direct antitumor effects [35].

### Cell cycle modulators

Upon the DNA damage induced by ionizing radiation, all eukaryotic cells activate protecting mechanisms associated with cell cycle arrest until the DNA damage is repaired and – in the case of too extensive damage – necrosis or apoptosis [36]. Radioprotectors may affect cell fate acting through both mechanisms: either promoting cell cycle arrest or inhibiting necrosis/apoptosis.

The apoptosis is largely a p53-dependent process and inhibition of p53-mediated apoptosis by chemicals results in increased radioresistance [37]. This can be achieved by direct inhibitors of p53 activity such as pifithrins [38] or by modulation of other important pro-apoptotic proteins. For instance, kukoamine increases the level of anti-apoptotic mediators (BCL2) and decreases the level of pro-apoptotic mediators (BAX and caspase-3) in a dose-dependent way [39]. Acteoside has been shown to inhibit expression of caspase 3, and thus to decrease apoptosis [40] in human skin fibroblasts. Similarly, atorvastatin down-regulates expression of caspase 3 [41]. Carvacrol is another compound with anti-apoptotic action shown in cultured human peripheral blood lymphocytes but the molecular mechanism of it is not clear [42]. Isofraxidin inhibits apoptosis in a p53-independent way via cytochrome C in addition to caspase 3 [43].

Apart from the down-regulating apoptotic answers, some radioprotective substances lead to cell cycle arrest. Resveratrol is one of the most well-studied examples of this group of compounds. It has effects on cyclin expression and induces S-phase arrest [44].

### DNA protectors

The radioprotectors can elicit their action by various mechanisms and DNA protection via decreasing DNA damage is among them. Moreover, the late effects of ionizing radiation are associated with DNA damage that can be visualized by persistent DNA Damage Response (DDR) foci and might be prevented by radioprotectors [45, 46]. Reduction of DNA damage might be reached by suppressing the formation of reactive species, detoxification of radiation-induced species, target stabilization, and enhancing the repair and recovery processes [22]. The chemical or biochemical consumption of oxygen can lead to hypoxia in cells and tissues. This may be one of the mechanisms by which sulphydryl compounds (RSH), which can undergo an oxidation reaction with molecular oxygen, result in radioprotection. Also, some interest has been drawn to the thiol-induced hypoxia caused by amifostine and cystaphos, which offer selectivity in protecting normal cells vs. tumor cells [47, 48].

Radioprotectors can also interact with cellular targets, like DNA, by forming mixed disulfides and prevent radiation damage by stabilizing the target. Several amino thiol radioprotectors, such as cysteamine and WR 1065, bind to DNA and their DNA binding is coupled with their radioprotective potency [47–50]. Since one of the most important molecular targets damaged by radiation is the genomic DNA of a cell, cells must repair these lessons. Thiols, such as glutathione and adeturon, may be involved in the repair of DNA single-strand breaks. Cells genetically deficient in GSH synthesis or cells in which GSH deficiency is produced by dl- Buthionine-sulfoximine or by hypoxia or misonidazole show a lack of DNA single-strand break repair [51–53].

The cellular defense mechanisms against radiation and chemical stresses elicit an early SOS response to damage and subsequent adaptation. The SOS response is required for eliminating lesions in DNA while the adaptation response is needed for restoring cellular metabolism and return to normal functioning. SOS repair plays a very important role in protecting the key molecular targets, which comprise the activation or synthesis of several proteins, DNA precursor synthesizing enzymes, and DNA precursors [54]. Drugs and chemicals, which stimulate or increase the activity of DNA precursor-synthesizing enzymes, such as ribonucleotide reductase, could function as radioprotectors. The administration of the drug indomethacin prior to radiation exposure to animals (mice and dogs) resulted in higher survival of animals from lethal doses of gamma-radiation [54]. All of these radioprotectors are listed in https://radioprotectors.org/home.

### Sunscreening agents

UV radiation has a broad spectrum, ranging from 40 to 400 nm, which is divided into Vacuum UV (40–190 nm), Far UV (190–220 nm), UVC (220–290 nm), UVB (290–320 nm), and UVA (320–400 nm), of which the latter two are medically important. UVA radiation is divided into two distinct subtypes: short-wave UVA (320–340 nm) and long-wave UVA (340–400 nm) [55].

Both UVA and UVB radiation can cause sunburn, photoaging reactions, erythema, and inflammation. Mechanisms that modulate UV-induced damage involve nuclear and mitochondrial DNA damage, generation of reactive oxygen species (ROS), immune suppression, lipid peroxidation (membrane damage), activation of matrix metalloproteinases [56].

Radioprotectors.org includes a set of compounds that exert protective effects against UV-spectrum irradiation and thus form a group termed “sunscreening agents”. This group can further be divided into two subgroups with different mechanisms: physical (inorganic) and chemical (organic) sunscreens. For organic compounds, the mechanism of action is based on their chemical structure involving an aromatic compound conjugated with a carbonyl group. This structure allows the absorption of high energy UV rays and the molecule switches to an excited state. As the molecule returns to the ground state, it releases the lower energy of longer wavelengths. [57] Such compounds as Avobenzone, Oxybenzone, Ecamsule, Octinoxate are FDA-approved components of topical sunscreens with different spectrums of absorption and various photostability. Octinoxate is identified as one of the potent UVB-absorbers [58], but is not photostable and degrades in the presence of sunlight after a short period of time, while Ecamsule, a very photostable product, acts as UVA-blocker. In animal studies, it prevented UVA-induced photoaging [59].

The mechanism of action of physical sunscreens, such as Zinc oxide, Titanium dioxide is based on the reflection and scattering of UV light. The reflective properties - reflective index, the size of the particles, the film thickness, and the dispersion of base determine the effectiveness of inorganic sunscreens [57]. Microfine zinc oxide has shown to be efficient against a wide range of UVA including UVA 1 (340 to 400 nm), but less efficient in blocking UVB, compared to Titanium oxide. Microfine titanium dioxide protects against UVA 2 (315-340 nm) and UVB but does not protect against UVA 1 [59]. Notably, both of these compounds have shown remarkable shielding properties against ionizing radiation and can also be classified as potential radioprotectors [60].

### Inductors of autophagy

Autophagy is the essential, regulated cellular mechanism that disassembles and degrades unnecessary or dysfunctional components. Further recycling of those components serves as an additional energy source under various stress conditions [61]. In recent years, autophagy became one of the crucial cellular events in the context of aging research. Pharmacological or genetic inhibition of autophagy promotes degenerative tissue changes, resembling those that occur during aging and also reduces the longevity-promoting effects of caloric restriction. Contrariwise, interventions that stimulate autophagy, increase lifespan in model organisms - notably, among all pharmacological manipulations MTORC1 inhibition is known to have the most dramatic effect [62, 63]. Activation of AMPK, another key autophagy regulator, triggers a number of cellular-protective mechanisms and prevents the hydrogen peroxide-induced dysregulation of the autophagic flux in senescent cells [64].

Autophagy is a generally cytoprotective (rather than a self-destructive) process. However, under certain conditions autophagy machinery is likely to be required for essential cell death [65]. In some cases, autophagy shares rather pro-senescent than anti-senescent features - once the cell comes into a senescent state, autophagy is likely to sustain its viability by reducing the level of overall metabolic stress. Under normal conditions, autophagy exerts anti-senescence effects. [66] Such dual nature of the autophagic process opens a perspective to use destructive autophagy properties to combat cancer and it’s a progression in some cases, by triggering autophagic cell death or senescence of malignant cells [67].

**Figure 2:**
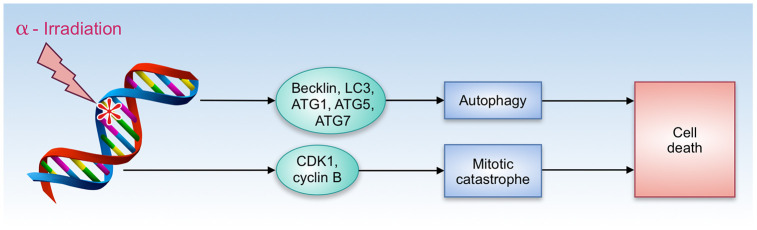
**Autophagy and cell death by α-radiation.**

A number of compounds that share both gero- and radioprotective properties have an ability to promote autophagy - understanding of how this feature contributes to radioresistance is important for further radioprotectors research and development. In a short-term period after irradiation, autophagy plays a positive role due to its cytoprotective properties. Autophagy protects the hematopoietic system from nuclear injury through modulation of DDR (DNA damage response) [68]. However, in the long-term perspective, the role of autophagy remains controversial. Malignant transformation of irradiated cells remains one of the most serious long-term consequences of radiation-induced damage. A number of studies have revealed that cancer cells rely on autophagy to gain radioresistance [69, 70]. On the other hand, irradiation has an ability to trigger autophagic cell death that involves Becklin, LC3, ATG1, ATG5, and ATG7 proteins (Figure 2) [71].

Remarkably, some radioprotective compounds such as Buthionine sulfoximine, Hoechst 33342, exert dual activity - enhance radioresistance in normal and radiosensitized transformed cells.

Due to the ability of some autophagy inductors to promote cancer cell apoptosis and display negative effects on cancer cell metabolism, natural compounds that can synergically work with chemotherapy agents have received certain attention in the field of cancer research. [72]. Such plant-derived components as Luteolin [73], Naringin [74], Caffeine [75] showed an inhibitory effect on tumor cell growth and enhanced apoptosis.

**Figure 3:**
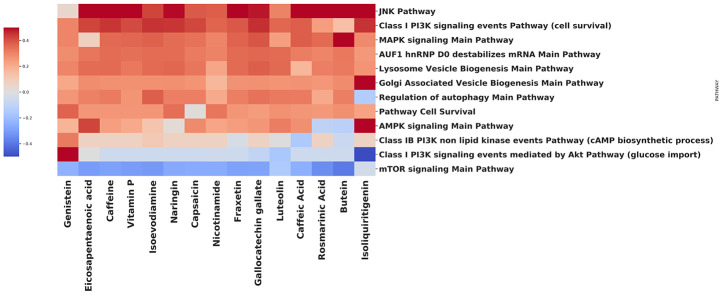
**Effect of compounds on autophagy-related pathways.**

We have performed an analysis to identify how various natural compounds, including those with gero- and radioprotective activities, modify the activity of the common autophagy-associated pathways (Figure 3). In a vast majority, the upregulation of AMPK signaling pathway and downregulation of mTOR signaling pathway was observed. Notable upregulation of pathways that are involved in lysosome vesicle biogenesis was also shown for most of the compounds. MAPK signaling pathway activation may be related to the mTORC1-MAPK feedback loop, which was observed both in cancer and normal cells [76]. In common, signaling pathway landscape induced by most of the compounds, identifies them as potent autophagy inductors.

Using the open database LINCS1000, we have collected gene expression profiles for each compound on the heatmap. In order to obtain the list of differentially expressed genes, data were processed using the R 'limma' package [77] Benjamini-Hochberg FDR adjustment was applied to the p-values [78]. The pathway-level analysis was performed using the iPANDA software suite [79]. Positive and negative iPANDA scores indicated up- and down-regulation of the pathway, respectively. The pathway database used for the analysis included 1856 annotated and manually curated signalling pathway maps from KEGG, Reactome, and NCI-PID and SA Biosciences collections [80–82].

### Comparison of radio- and geroprotectors databases

There is a substantial intersection between aging and radiation-induced damage [83]. Multiple radiation-induced conditions are classified as diseases [84], and aging and radiation-accelerated aging may be classified as diseases [85]. Significant crosstalk between the mechanisms underlying the radiation protection and geroprotection is also notable [19, 86]. Many of the compounds that can be found in https://radioprotectors.org/home can be also found in the known databases of geroprotectors [87, 88]. In total, 66 substances included in the present database of radioprotectors also show geroprotective activity being listed in the Geroprotectors.org database [88]. These compounds are shown in Table 1. The functional similarity between geroprotectors and radioprotectors is partially due to the similar nature of negative effects on DNA imposed by radiation and developed during aging. Damages of the genetic material gradually accumulate throughout life, as the effectiveness of the repair systems and the ability of cells to neutralize genotoxic factors decrease. The death and senescence of cells, leading to fibrosis and chronic inflammatory processes, as well as a decrease of the stem cell pool and their malignant transformation under conditions of genotoxic stress, are key events in the aging process [89–91].

**Table 1:** Chemical compounds with combined gero- (according to Geroprotectors.org) and radio-protective activity.

**Compound name**	
Amifostine, Lithium chloride, Vitamin D3, Kanamycin, 2,4-dinitrophenol, Ellagic acid, Catechin, Carbonyl, Cyanide M-Chlorophenyl Hydrazone (CCCP), Glycerol, Deprenyl, Trichostatin A, Cysteamine, Quercetin, Fisetin, 4'-o-methyl epicatechin, Cyclosporin A, Valproic acid, Metformin, Rosmarinic acid, Rapamycin, Ibuprofen, Resveratrol, Simvastatin, Caffeine, Nitrendipine, Euk-134, Caffeic acid, Indirubin, 1,2,3,4,6-Penta-O-Galloyl-B-D-Glucose (PGG), Carnosine, Dimethyl Sulfoxide (DMSO), Ly294002, 4- phenylbutyrate, Beta- estradiol, Epigallocatechin Gallate, Minocycline, 10-Hydroxy-2-decenoic acid, Baicalein, Gallic acid, N-acetyl-L-cysteine, Genistein, Aspirin (Acetylsalicylic acid, ASA), Quercetin-3-O-Glucoside, Enalapril, Α-Lipoic Acid, Celecoxib, Ursolic acid, Curcumin, Kaempferol, Melatonin, Ascorbic acid, Polydatin, Sodium Butyrate, Spermidine, 2-mercaptoethanol, Maltose, Trehalose, Cyproterone acetate, Fenofibrate	
Glutatione	The effect is ambiguous [107]
Pioglitazone	Effect was shown for a derivative [108]
Butylated hydroxytoluene (BHT)	The effect was shown for S. cerevisiae but not for cell cultures [109]
Fullerene C60	Effect was shown for a derivative [110]
Doxycycline	In combination with valproic acid [111]
Fumarate	Effect was shown for a derivative [112]
Nitroflurbiprofen	Effect was shown for a derivative [113]

Ultraviolet radiation is considered one of the key factors in skin aging as well as an inducer of the above-mentioned processes. At the same time, ionizing radiation rapidly causes numerous and often unrepairable lesions (i.e., double-strand DNA breaks), leading to a vast cell death, primarily of the cells with a high proliferative index. A critical event is the almost complete inhibition of hematopoiesis and depletion of the bone marrow stem cells. Radiation causes the development of a senescent phenotype as a protective mechanism against a possible malignant transformation. Thus, the processes of aging and irradiation-induced changes are closely related at molecular and cellular levels [19]. Substances possessing gero- and radioprotective properties can exhibit similar protective effects (for example, act as antioxidants and reduce the number of free radicals formed both naturally in the processes of cell metabolism and those resulting from radiolysis), as well as affect the same signaling pathways leading to positive effects [92, 93].

Development of new effective drugs against aging and radiation-induced aging is an ambitious but at the same time pleading task. Several approaches can be applied to solve the problem including pathway analysis and searching for new targets [19, 93–95], searching for possible biomarkers for both aging and radiation exposure [96–104] and even generation of new chemical compounds [105, 106]. However, all of them rely on the availability of profoundly annotated data about chemical compounds with radioprotective effects and their molecular modes of action. The present curated database of radioprotectors will become a convenient onset for the development of medicines against radiation-induced damage and aging following both the structure-based and ligand-based approaches.
